# A real-time smart sensing system for automatic localization and recognition of vegetable plants for weed control

**DOI:** 10.3389/fpls.2023.1133969

**Published:** 2023-03-27

**Authors:** Jia-Le Li, Wen-Hao Su, He-Yi Zhang, Yankun Peng

**Affiliations:** College of Engineering, China Agricultural University, Haidian, Beijing, China

**Keywords:** crop signalling, computer vision, plant identification, automated weeding, precision agriculture

## Abstract

Tomato is a globally grown vegetable crop with high economic and nutritional values. Tomato production is being threatened by weeds. This effect is more pronounced in the early stages of tomato plant growth. Thus weed management in the early stages of tomato plant growth is very critical. The increasing labor cost of manual weeding and the negative impact on human health and the environment caused by the overuse of herbicides are driving the development of smart weeders. The core task that needs to be addressed in developing a smart weeder is to accurately distinguish vegetable crops from weeds in real time. In this study, a new approach is proposed to locate tomato and pakchoi plants in real time based on an integrated sensing system consisting of camera and color mark sensors. The selection scheme of reference, color, area, and category of plant labels for sensor identification was examined. The impact of the number of sensors and the size of the signal tolerance region on the system recognition accuracy was also evaluated. The experimental results demonstrated that the color mark sensor using the main stem of tomato as the reference exhibited higher performance than that of pakchoi in identifying the plant labels. The scheme of applying white topical markers on the lower main stem of the tomato plant is optimal. The effectiveness of the six sensors used by the system to detect plant labels was demonstrated. The computer vision algorithm proposed in this study was specially developed for the sensing system, yielding the highest overall accuracy of 95.19% for tomato and pakchoi localization. The proposed sensor-based system is highly accurate and reliable for automatic localization of vegetable plants for weed control in real time.

## 1 Introduction

Tomato (Solanum lycopersicon) is one of the major commercial vegetable crops in the world (Johansen et al., 2019). Tomato is considered to be beneficial to human health, and they are widely grown and consumed all over the world (Chaudhary et al., 2018), with great commercial and economic values (Brunetti et al., 2019). However, the production of this crop is being threatened by weeds (Qasem, 2019). The competition between weeds and crops for resources such as sunlight and nutrients is one of the main factors affecting crop yields (Hamuda et al., 2016). Early weed control is one of the most important initiatives to prevent significant crop yield losses (Su et al., 2020a).

The most prominent method of weed management is herbicide spraying throughout the crop area (Pérez-Ortiz et al., 2016), but large-scale spraying of herbicides has a negative impact on the environment (Rodrigo et al., 2014). Moreover, incomplete degradability of herbicides can leave residues in plants (Rojas et al., 2017), creating health risks if the products with herbicide residues were consumed by humans and animals (Manh et al., 2001). Additionally, available herbicides for vegetable crops are very limited (Westwood et al., 2018). As a conventional weeding method, mechanical cultivating is not only able to loosen the soil but also can remove weeds between rows (Wang et al., 2019). Nevertheless, this method does not work for intro-row weeds. Intro-row weeds have traditionally been removed by manual weeding. However, manual weeding is inefficient and particularly prone to errors (Slaughter et al., 2008). This approach is increasingly costly, unsustainable and uneconomical in the long run due to labor shortages (Fennimore et al., 2017).

With the increase of the global population and the improvement of living standards, the demand for healthy food continues increasing. Predicting biomass through various types of sensing technologies has become the focus of precision agriculture and smart farming in recent years (Johansen et al., 2020). Precision weeding is considered as one of the most important measures for sustainable vegetable production (Raja et al., 2020a). There is a need to develop an efficient autonomous weeding robot that can intelligently identify weeds and enable precise herbicide application. The primary task of developing a smart weeder is to identify the vegetable crops and weeds accurately and timely.

Detection methods based on characteristics of crop plants and weeds such as color (Gupta and Ibaraki, 2014), size (Lamm et al., 2002), and spectral reflectance (Borregaard et al., 2000) have been proposed to classify crops and weeds. Blasco et al. (2002) developed a robotic system for weed control in transplanted lettuce, which identified the plants with the accuracies of 84% and 99% for weeds and lettuce based on the plant size, respectively. But the probability of misidentification increases significantly when weeds and crops are of similar size. Vrindts et al. (2002) developed a hyperspectral machine vision system to identify weeds in sugar beet fields. However, their method could not be directly used for the real-time detection of the plants. Lin et al. (2017) established a method combining plant spectrum, shape and texture features to discriminate maize and weed with over 95% accuracy, but the spatial location of the plant was not considered in their study. With the development of computer vision technology, more sophisticated object detection methods based on imaging and machine learning have been developed to distinguish crops from weeds (Hall et al., 2017; Lottes et al., 2017; Milioto et al., 2018; Tang et al., 2018; Rehman et al., 2019; Li et al., 2022). Ahmed et al. (2012) proposed a support vector machine method to classify crops and weeds, which achieved above 97% accuracy. dos Santos Ferreira et al. (2017) developed a ConvNets-based software for detecting weeds in soybean, yielding an average accuracy of 99.5%. Subeesh et al. (2022) applied the InceptionV3 model to identify weeds in sweet peppers with an accuracy of 97.7%. However, the above methods need to obtain a large number of samples in advance to label them accurately for model training.

The up-to-date technology called crop signaling was proposed to simplify weed identification (Raja et al., 2019b). This technology creates a machine-readable signal on plants that allows the marked crops to be readily identified by a computer vision system. Crop signaling technology has been successfully used to identify different target plants in weeds (Nguyen et al., 2017; Vuong et al., 2017; Raja et al., 2019a; Su, 2020). For example, Raja et al. (2020b) developed a device containing two cameras to automatically detect lettuce and weeds based on crop signaling. The classification accuracies of lettuce plants and weeds were 99.75% and 83.74%, respectively. However, the use of two cameras to obtain the location information of plants increased the complexity of image processing. In another study, Su et al. (2020b) applied systemic signaling markers to tomato and lettuce plants, allowing the treated plants to be efficiently detected by a single imaging system. Although their technology enabled the detection of exogenous signals applied to crops, the development of the equipment that can be used for the online detection of plant markers has not yet been reported.

In this study, an intelligent sensing system equipped with six color mark sensors and a color camera is expected to be developed for automatic identifications of weed and tomato plants. The color mark sensor is a photoelectric sensor used to quickly detect a specific color label based on the difference between the gray value of the target label and the reference. The color mark sensor is a reverse device, which realizes detection by receiving and analyzing the scattered light of the detected object. The detection principle of the color mark sensor mainly involves three steps. First, the color mark sensor emits monochromatic light (or white light) with the same intensity to the surface of the measured label. Then, it receives the diffuse reflection from the surface of the measured label. Finally, the label is identified according to whether the intensity of the diffuse reflection is consistent with the preset reference value. Prior to detection, the color mark sensor detects and records the amount of the diffuse reflection from the reference and target, respectively. This amount is used as the preset reference value. When an equivalent amount is detected, the object is the desired target. Although the color mark sensor has been widely used in packaging, printing, spinning and other industries (Yang et al., 2009), it has not been used in plant recognition yet.

The captured images were converted from red-green-blue (RGB) space to hue-saturation-value (HSV) space for color segmentation in this study. The reason is that the images acquired in the natural environment are sensitive to illumination variation, and all three components of the RGB space are closely related to illumination. If the illumination of the image changes, it may not be possible to completely separate the plant pixels from the background using RGB space (Hamuda et al., 2016). In contrast, the HSV space is robust to illumination changes (Siogkas and Dermatas, 2006). Additionally, HSV space is more in line with human color perception (Saxe and Foulds, 1996). This makes HSV space more suitable for segmenting plants and backgrounds (soil and residues) and has been widely used in various computer vision applications (Huang et al., 2015).

The main objective of this study was to develop a novel detection method for smart differentiation of tomato plants from packchoi (Brassica chinensis L.) plants. The specific objectives were 1) developing a smart sensing system containing several color mark sensors and a color digital camera, 2) comparing experimental results of arranging color mark sensors and plant labels in different schemes to determine the best combination. 3) evaluating effects of the signal tolerance zone size on the performance of the system in locating weeds and tomato plants to determine the final solution. As far as we know, this was the first study to realize real-time identification and localization of tomato from background plants using a composite sensing system developed by combining computer vision and color mark sensing.

## 2 Materials and methods

### 2.1 Plants and labels

A series of experiments were carried out in the laboratory of China Agricultural University. 3 week-old tomato plants (height: 15 - 20 cm, the maximum width of the canopy: 10 - 20 cm) were selected as crop samples (300 plants). 1 week-old pakchoi plants (height: 2 - 10 cm, the maximum width of the canopy: 3 - 8 cm) were selected and regarded as control plant (weed) samples (1114 plants). Both plant seedlings purchased from commercial nursery were transplanted into plastic pots filled with moist soil and grown in a controlled environment (temperature = 18–22°C, relative air humidity = 46–50%). Pakchoi seedlings were planted between tomato plants. In this study, tomato plants were labeled by plant labels and the control plants without plant labels were considered as weeds in this study.

**Figure 1:**
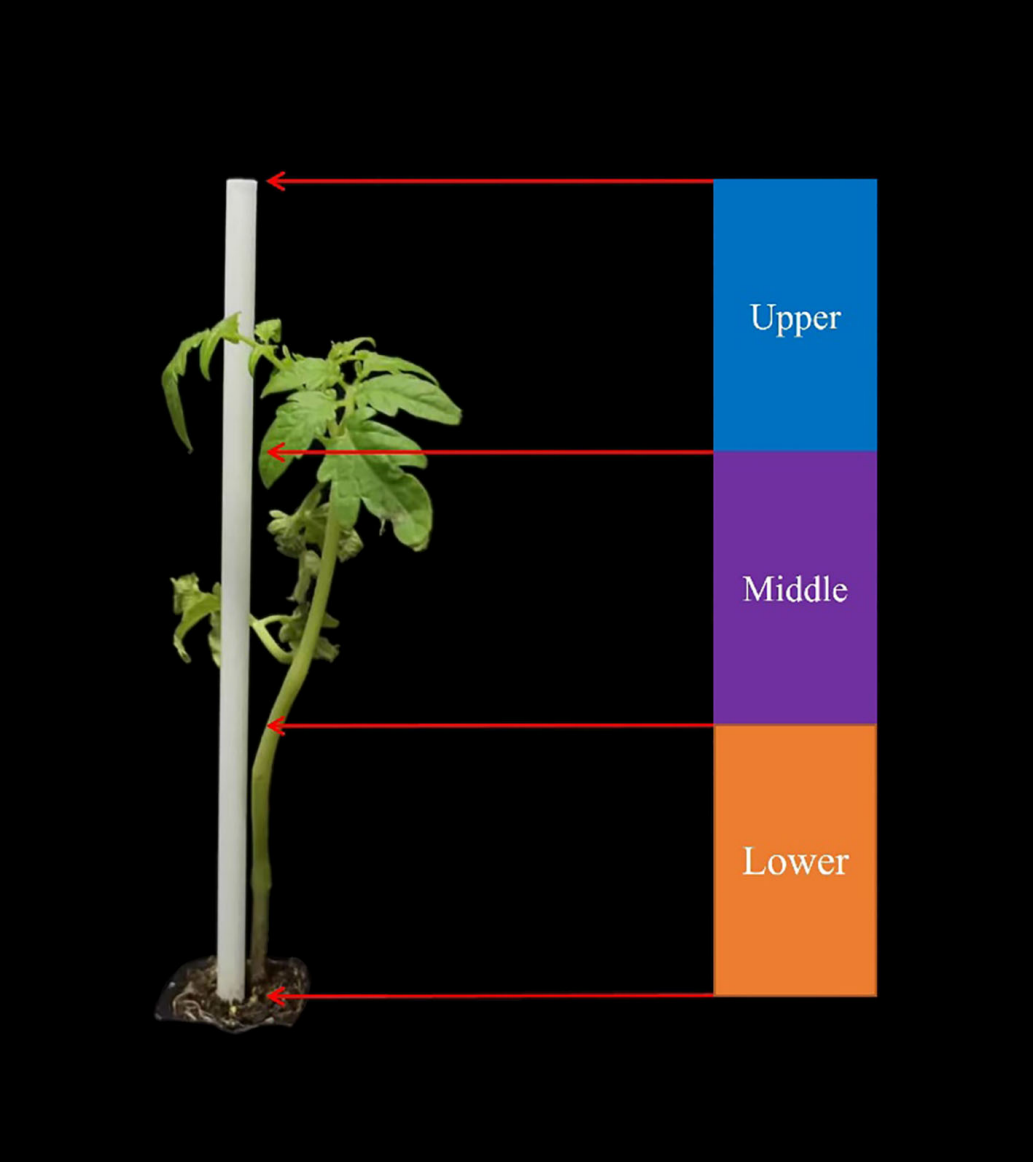
Physical plant label (white straw) and tomato plant.

**Figure 2:**
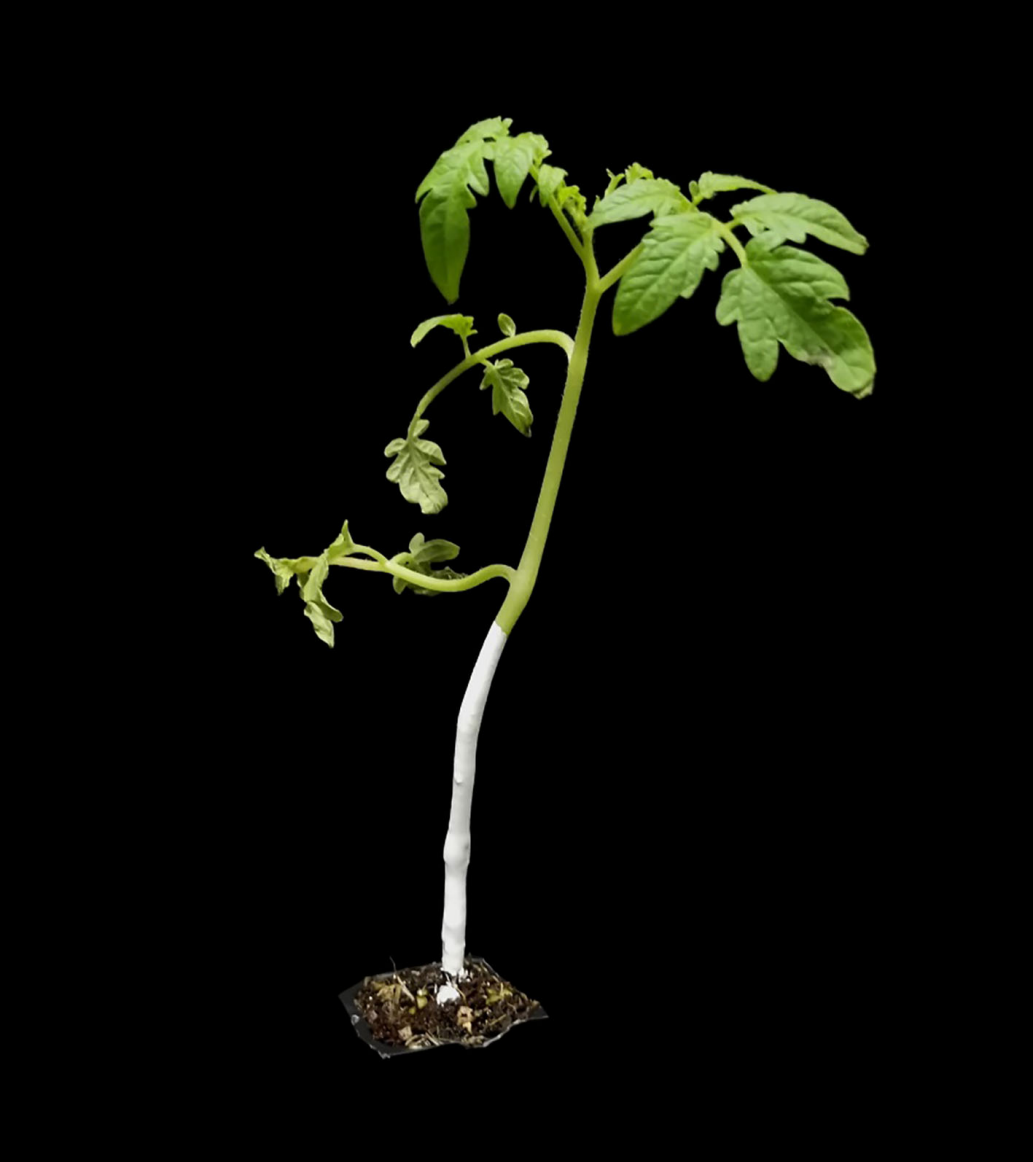
Topical marker (white paint) and tomato plant.

Two types of signaling markers including physical labels and topical markers were selected as the plant labels. The physical label (red, white and green) is an environmentally friendly straw (23 cm in length) made of polylactic acid that can be degraded by microorganisms. When the physical plant label was used to label a tomato plant, it was inserted into the soil near the roots of the tomato plant and attached to the main stem of the tomato plant, as shown in **Figure 1**. The above-ground part of the physical label is divided into three areas, including the lower part (0 - 6cm), the middle part (6 - 12cm) and the upper part (12 - 18cm). The topical marker (white) is an environmentally friendly paint. When the topical marker was used to label a tomato plant, it was applied to the lower main stem of the tomato plant, as shown in **Figure 2**. Tomato plants were successively marked with different physical labels (red, white, or green) and white topical markers.

**Figure 3:**
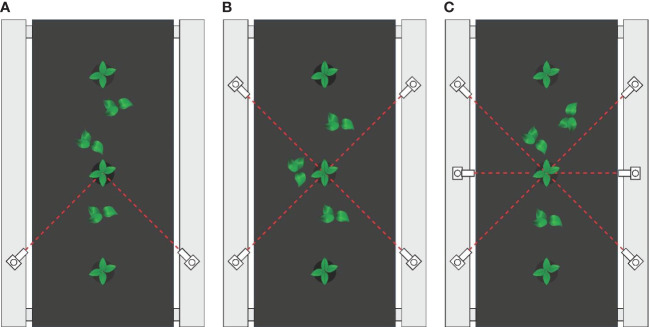
**(A)** 2 color mark sensors were applied, **(B)** 4 color mark sensors were applied, **(C)** 6 color mark sensors were applied.

Tomato plants with plant labels were placed on a conveyor belt and detected by a color mark sensor 20 cm away from them. Weed stems and tomato stems were successively set as the reference for color mark sensor to determine the best reference for the color mark sensors and the best color for the plant labels. Three areas of physical plant labels and topical plant labels were detected by adjusting the mounting height of the sensor to determine the most suitable detection area. Immediately afterwards, Tomato plants marked with the topical marker (1-6 weed samples were randomly distributed around each tomato plant) was detected by combinations of different number of color mark sensors (2, 4 and 6, as shown in **Figure 3**) to determine the optimal number of color mark sensors for this system. Finally, the size of the signal circular tolerance zone (r = 2.5mm, r = 5mm, r = 7.5mm, r = 10mm and r = 12.5mm) on the performance of the system was evaluated with tomato plants and weeds.

### 2.2 Plant sensing system

**Figure 4:**
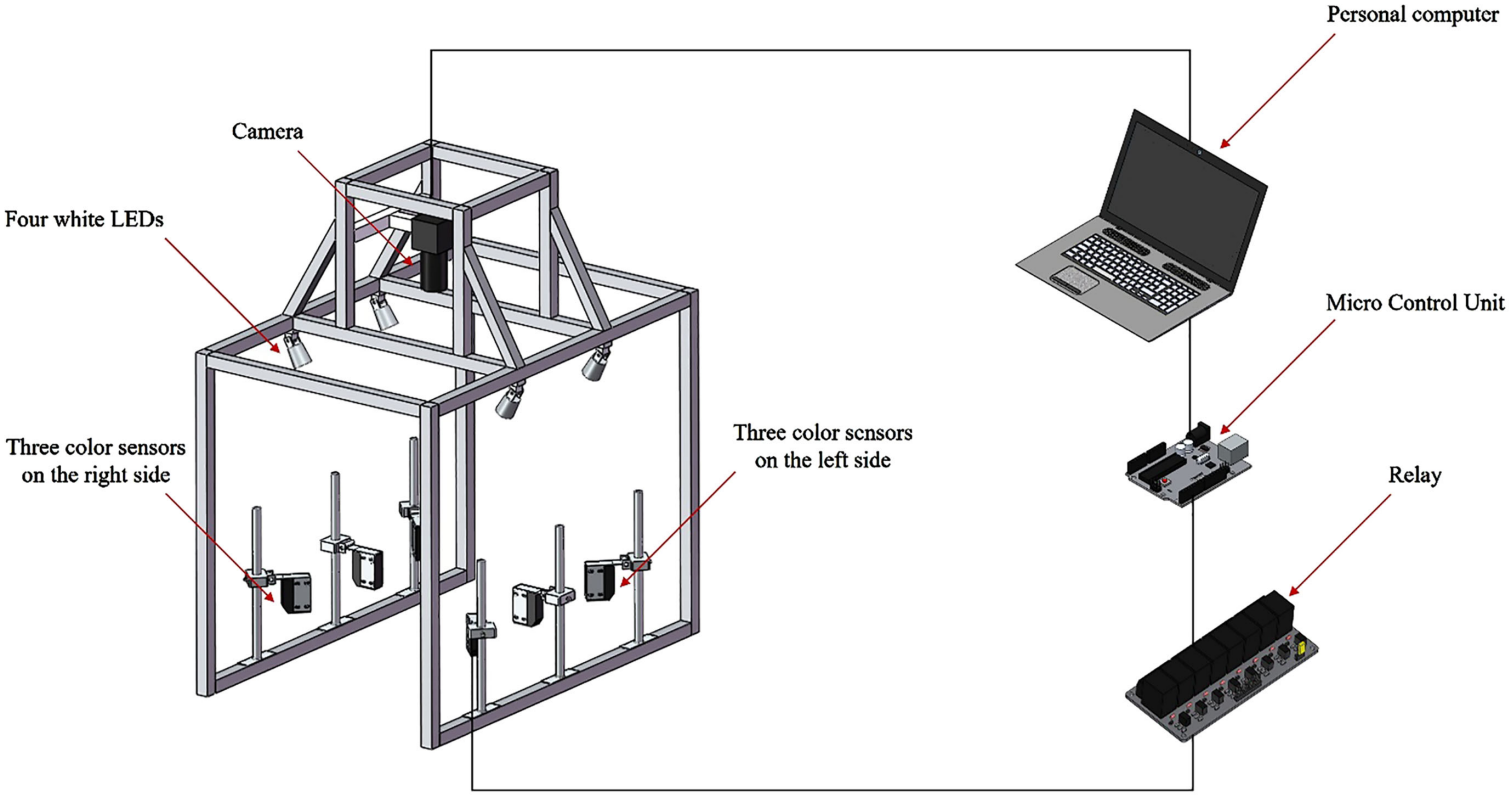
The mechanical structure of the detection device.

The sensing system consists of an adjustable voltage power, a relay, a micro control unit, a personal computer and an enclosed chamber with black vinyl walls. The chamber mainly included a color digital camera, six color mark sensors, four white light emitting diodes (LEDs), as shown in **Figure 4**. The camera was placed on the vertical centerline between the middle color mark sensors at a height from the plant so that the top view of crop plants was visible. The camera was controlled by the personal computer and was used to capture images of tomato plants and the weeds around them. The color mark sensors emitting red light with a long emission distance of 30 cm were used to detect plant labels. Two middle color mark sensors were positioned parallel to the travel direction. The other color mark sensors were positioned at an angle of 45° relative to the middle sensors. The combination of six color mark sensors allowed the sensing system to cope with situations where the signal was obscured by weeds. The system could detect plant labels as long as two sensors that not on the same detection line were not blocked. The white LEDs were placed below the camera around the upper part of the chamber to provide even illumination for imaging the plants. An adjustable voltage power supply was used to control the brightness of the LEDs. Each color mark sensor was equipped with a relay. When the color mark sensor detected the plant label, it sent a signal to the relay immediately. The microcontroller was connected to all the relays to capture the signals of all the color mark sensors simultaneously. The code received by the microcontroller was interpreted by the software and transmitted to the personal computer to calculate the location of the plant label. The entire system was mounted on a conveyor belt with a speed of 0.91km/h.

### 2.3 Plant classification algorithm

**Figure 5:**
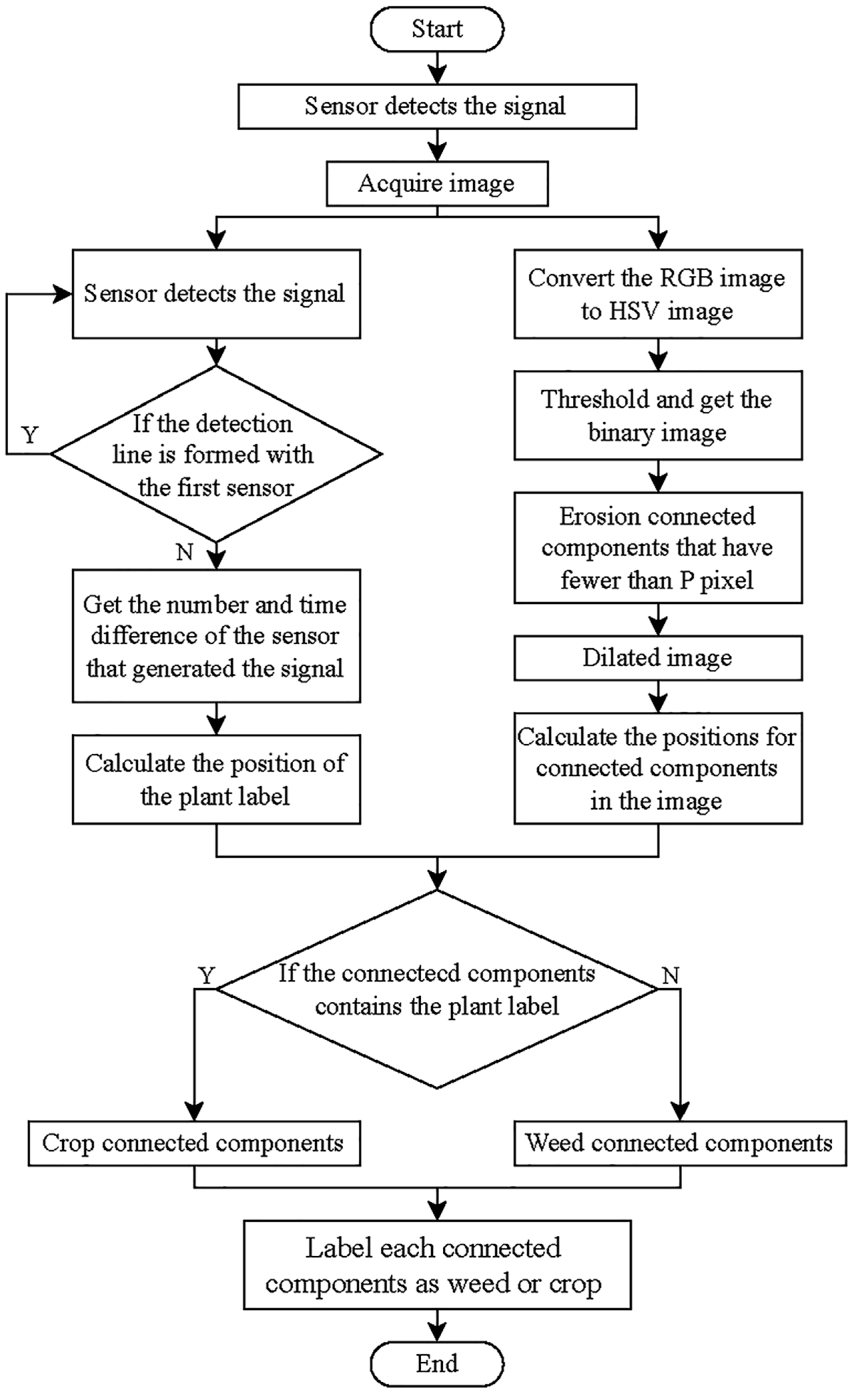
Flow chart of the machine vision algorithm for weeds and tomato plants classification.

**Figure 6:**
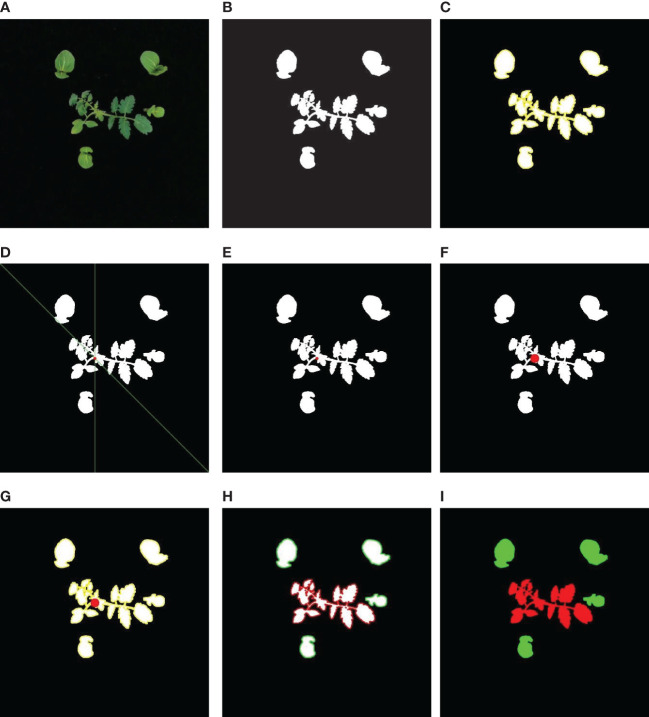
**(A)** Image of tomato plant and weeds captured by the system, **(B)** binary image after morphological operations, **(C)** binary image with plants contours (yellow), **(D)** position of the crop signal in the RGB image, **(E)** position of crop signal in the binary image, **(F)** crop signal tolerance bands in the binary image, **(G)** the contours of plants (yellow) and the crop signal tolerance bands (red) in the binary image, **(H)** the contours of tomato plant (red) and the contours of weeds (green), **(I)** pixel mapping of weeds (green) and crop plants (red).


An algorithm was especially developed to acquire images of tomato and packchoi plants based on the signals of the smart sensing system and classify them. The ultimate purpose of image processing was to map weeds. The flow chart of the algorithm is shown in **Figure 5**. The color mark sensors detect passing tomato plants with plant labels while the conveyor is running. When the color mark sensor detects a plant label, the image was captured by the camera. With white LED illumination, the camera’s exposure was set to -6 to obtain sufficient image intensity while eliminating motion blur from the conveyor belt. The image captured by the camera was shown in **Figure 6A**. Based on the RGB image obtained under controlled illumination, the hue (H), saturation (S) and luminance (V) values were calculated for each pixel and the HSV space model of the image was generated. The HSV space was used for color partitioning of all plants (both tomato plants and weeds) from the background. The threshold of color segmentation was determined based on the appearance of plant colors in the image. The rules for color segmentation were shown in Equation 1.

(1) $${\text{R}}_{\text{t}}(\text{x},\text{y})=\{\begin{array}{c} 255\;\begin{array}{c} \text{if}{\left[{\text{T}}_{h}\right]}_{\text{low}}\leq {\left[{\text{O}}_{\text{hsv}}(\text{x},\text{y})\right]}_{\text{h}}\leq {\left[{\text{T}}_{\text{h}}\right]}_{\text{high}}\text{and}\\ {\left[{\text{T}}_{\text{s}}\right]}_{\text{low}}\leq {\left[{\text{O}}_{\text{hsv}}(\text{x},\text{y})\right]}_{\text{s}}\leq {\left[{\text{T}}_{\text{s}}\right]}_{\text{high}}\text{and}\\ {\left[{\text{T}}_{\text{v}}\right]}_{\text{low}}\leq {\left[{\text{O}}_{\text{hsv}}(\text{x},\text{y})\right]}_{\text{v}}\leq {\left[{\text{T}}_{\text{v}}\right]}_{\text{high}} \end{array}\\ 0\text{ otherwise } \end{array}$$

Where *O_hsv_* (*x*, *y*) represents the mapping of the captured original image in the HSV space model. [*O_hsv_* (*x*, *y*)]*_h,s,v_* are the hue, saturation and value of *O_hsv_* (*x*, *y*), respectively. [*T_h,s,v_*]*_low_*, are the low thresholds for the H, S and V channel, respectively. Correspondingly, [*T_h,s,v_*]*_high_*, are the high thresholds for the H, S and V channel, respectively. The thresholds values were selected by pre-experiments. Some images were acquired before the formal experiments and these they were binarized with different thresholds. The most suitable thresholds were selected by comparing the results. The thresholds used in the research were [*T_h_*]*_low_* = 10, [*T_h_*]*_high_* = 120, [*T_s_*]*_low_* = 10, [*T_s_*]*_high_* = 255,[*T_v_*]*_low_* = 10, [*T_v_*]*_high_* = 255. *R_t_* (*x*, *y*) represents the result of color segmentation, a binary image consisting of pixel points in the threshold range.

A 3×3 square structuring element was used for the erosion operation in the binary image. After, tomato plants and weeds in the image were separated from the background. The processed image was then dilated with the same structuring elements to restore the connectivity of the connected domains while maintaining the consistency of the object size. The binary image after morphological operations is shown in **Figure 6B**. The contour of each connected component was measured in the image and plotted in yellow, as shown in **Figure 6C**.

As the plant labels pass through the detection area of the sensor combination, they will be detected as positive by multiple sensors. The signals generated by two sensors that are not on the same detection line are combined into a group of available signals. The sensor numbers that make up the available signal are sent to a personal computer as the basis for calculating the positions of the plant labels in the images. According to the data output by the sensor and the moving speed of the device, the detection lines of the sensor were drawn in the captured image, as shown in **Figure 6D**. The Cartesian coordinate system was established with the point at the upper left of the image as the origin. The detection lines for each sensor were represented by a function equation. The intersection of the sensor detection lines was the position of the plant label in the image, which was plotted on the binary image, as shown in **Figure 6E**. The rules for calculating the coordinates of the plant labels were shown in Equation 2. Then, a circular tolerance zone was determined. All pixels within the tolerance zone were considered as plant labels, as shown in **Figure 6F**.


(2) $$\begin{array}{l} \text{if }(\text{Ser}\_1\;==\;1\text{ or Ser}\_1\;==\;4)\;\&\;\left(\text{Ser}\_2\;==\;2\text{ or Ser}\_2\;==\;5\right):\\ \;\;\;\;\;\;\;\;\text{point}\_\text{signal }=\;\left(\text{round}\left(360\;-\text{ dt }*\text{ v}\right),\text{ round}\left(360\;-\text{ dt }*\text{ v}\right)\right)\\ \text{elif }(\text{Ser}\_1\;==\;1\text{ or Ser}\_1\;==\;4)\;\&\;\left(\text{Ser}\_2\;==\;3\text{ or Ser}\_2\;==\;6\right):\\ \;\;\;\;\;\;\;\text{point}\_\text{signal }=\;\left(\text{round}\left(360\;-\;\left(\text{dt }*\text{ v}\right)\;/\;2\right),\text{ round}\left(360\;-\;\left(\text{dt }*\text{ v}\right)\;/\;2\right)\right)\\ \text{elif }(\text{Ser}\_1\;==\;2\text{ or Ser}\_1\;==\;5)\;\&\;\left(\text{Ser}\_2\;==1\text{ or Ser}\_2\;==\;4\right):\\ \;\;\;\;\;\;\;\text{point}\_\text{signal }=\;\left(360,\text{ round}\left(360\;+\text{ dt }*\text{ v}\right)\right)\\ \text{elif }(\text{Ser}\_1\;==\;2\text{ or Ser}\_1\;==\;5)\;\&\;\left(\text{Ser}\_2\;==\;3\text{ or Ser}\_2\;==\;6\right):\\ \;\;\;\;\;\;\;\;\text{point}\_\text{signal }=\;\left(360,\text{ round}\left(360\;-\text{ dt }*\text{ v}\right)\right)\\ \text{elif }(\text{Ser}\_1\;==\;3\text{ or Ser}\_1\;==\;6)\;\&\;\left(\text{Ser}\_2\;==\;1\text{ or Ser}\_2\;==\;4\right):\\ \;\;\;\;\;\;\;\;\text{point}\_\text{signal }=\;\left(\text{round}\left(360\;-\;\left(\text{dt }*\text{ v}\right)\;/\;2\right),\text{ round}\left(360\;+\;\left(\text{dt }*\text{ v}\right)\;/\;2\right)\right)\\ \text{elif }(\text{Ser}\_1\;==\;3\text{ or Ser}\_1\;==\;6)\;\&\;\left(\text{Ser}\_2\;==\;2\text{ or Ser}\_2\;==\;5\right):\\ \;\;\;\;\;\;\;\;\text{point}\_\text{signal }=\;\left(\text{round}\left(360\;-\text{ dt }*\text{ v}\right),\text{ round}\left(360\;+\text{ dt }*\text{ v}\right)\right) \end{array}$$

The tolerance zone of the plant labels was integrated with the plant contour, as shown in **Figure 6G**. The pixel coordinates of each connected area in the image were retrieved. Then, the connected region with the plant labels was considered as tomato plants, which was marked in red. In this study, only one tomato plant was included in each image. The connected region without plant labels was considered as weeds, which was marked in green, as shown in **Figure 6H**. Based on detection results, the prescription map for the distribution of weeds and crop plants was drawn as shown in **Figure 6I**.

## 3 Results

### 3.1 Selection of references for color mark sensor

The effect of two references (the packchoi stem and tomato stem) for color mark sensors on the detection accuracy of physical plant labels of three colors (red, white, and green) was evaluated. As shown in **Table 1**, the average accuracy of the color mark sensor for plant label recognition was 31.00% when the weed stem was used as the reference. When the tomato stem was used as the reference, 48.89% of the plant labels were successfully identified overall. As can be seen, the color mark sensor with the tomato stem as the reference achieved better recognition performance on plant labels in red or white than that using the weed stem as the reference. Thus, the tomato stem was selected as the reference for color mark sensor for further study.

**Table 1:** The results of detecting different physical plant labels based on different reference.

Reference	Color of physical plant label	Samples	Correct detection	Accuracy	Average accuracy
Weed stem	Red	300	73	24.33%	31.00%
	White	300	206	68.67%	
	Green	300	0	0.00%	
Tomato stem	Red	300	166	55.33%	48.89%
	White	300	274	91.33%	
	Green	300	0	0.00%	

### 3.2 Determination of optimal plant label color

The optimal plant label color was selected using the tomato stem as the sensor reference. The detectability of plant labels in red, white, and green was evaluated. As shown in **Table 1**, the detection accuracy of red, white, and green plant labels was 55.33%, 91.33% and 0.00%, respectively. Compared to green plant labels, both red and white plant labels can be detected by the color mark sensor. Although the red labels were able to be used as plant labels, the color mark sensor was more sensitive to the white labels (91.33%). This shows that the white plant label is the best label recognized by the color mark sensor, followed by the red plant label. Thus, the white plant labels were selected for further study.

### 3.3 Determination of optimal detection area and category for plant label

**Figure 7:**
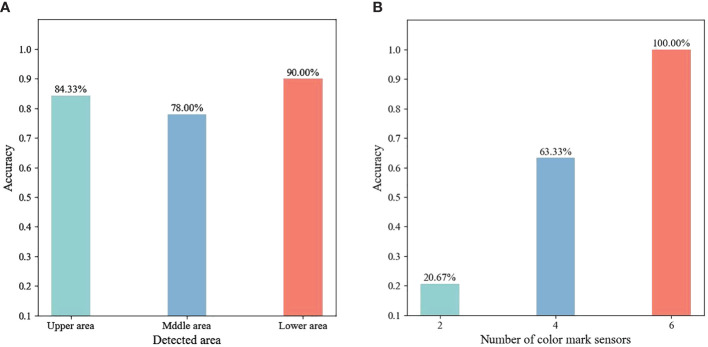
**(A)** Accuracy of detecting different areas, **(B)**accuracy of different number of sensors being applied.

The detectability of the color mark sensor to the upper, middle and lower regions of the white plant label was analyzed. The feasibility of detecting the three areas of the plant label in the horizontal direction by sensors placed at three heights (lower area: 0-6cm, middle area: 6-12cm and upper area: 12-18cm) was investigated. As shown in **Figure 7A**, the color mark sensors identify the lower area of the plant label with an accuracy of 90.00%. In contrast, the color mark sensor has lower detection accuracy for the middle and upper areas of the plant labels. This indicated that the lower area of the plant label was the optimal detection area. In order to eliminate the occlusion of plant labels by tomato stems, a new topical label was used in this study, in which white topical markers were applied directly to the tomato stems instead of the use of white physical labels. The sensor detected the white topical marker on the tomato stem with 100% accuracy. Therefore, the white topical marker was chosen as the most appropriate plant label for weed and tomato localization.

### 3.4 Determination of optimal number for color mark sensor

The effect of the number of selected sensors on the recognition of white topical marker was evaluated. Several weeds were randomly placed around each tomato plant. According to the algorithm developed in this study, the detection of a plant label was considered valid only if it was detected by two or more sensors that were not on the same symmetrical centerline. As shown in **Figure 7B**, the detection accuracy for plant labels increases as the number of sensors increases from 2 to 6. When 2 color mark sensors were used to detect plant labels, only 20.67% of the plant labels were successfully detected. When 4 color mark sensors work together, 63.33% of the plant labels were effectively detected by this sensing system. When the number of color mark sensors increased to 6, the sensing system detected all plant labels. Therefore, the combination of six color mark sensors was considered as the best solution for the number of sensors in the system.

### 3.5 Effect of the size of tolerance zone on plant localization

The effect of the size of the tolerance zone of estimated plant label locations on plant identification was evaluated. Five different sizes of circular tolerance zones (r = 2.5 mm, r = 5 mm, r = 7.5 mm, r = 10 mm and r = 12.5 mm) were set up as a basis for evaluating plant locations. As the radius of the tolerance zone increased from 2.5 mm to 12.5 mm, the accuracy of identifying tomato plants increased from 89.00% to 100.00%, while the accuracy of identifying weeds decreased from 95.42% to 93.72%, as shown in **Table 2**. To quantitatively evaluate all detected plants, the overall accuracy was calculated as shown in Equation 3.

**Table 2:** The results of detecting tomato plants based on different radius of the tolerance zone.

Radius of the tolerance zone	Detection object	Samples	Correct detection	Accuracy	Overall accuracy (%)
2.5mm	Tomato plants	300	267	89.00%	94.06%
	Weeds	1114	1063	95.42%	
5mm	Tomato plants	300	291	97.00%	95.12%
	Weeds	1114	1054	94.61%	
7.5mm	Tomato plants	300	294	98.00%	95.19%
	Weeds	1114	1052	94.435	
10mm	Tomato plants	300	295	98.33%	95.12%
	Weeds	1114	1050	94.25%	
12.5mm	Tomato plants	300	300	100.00%	95.05%
	Weeds	1114	1044	93.72%	


(3) $$\text{Overall accuracy}=\frac{\text{Tomato plants correctly detection}+\text{weeds correctly detection}}{\text{Tomato plants samples}+\text{weeds samples}}$$

**Figure 8:**
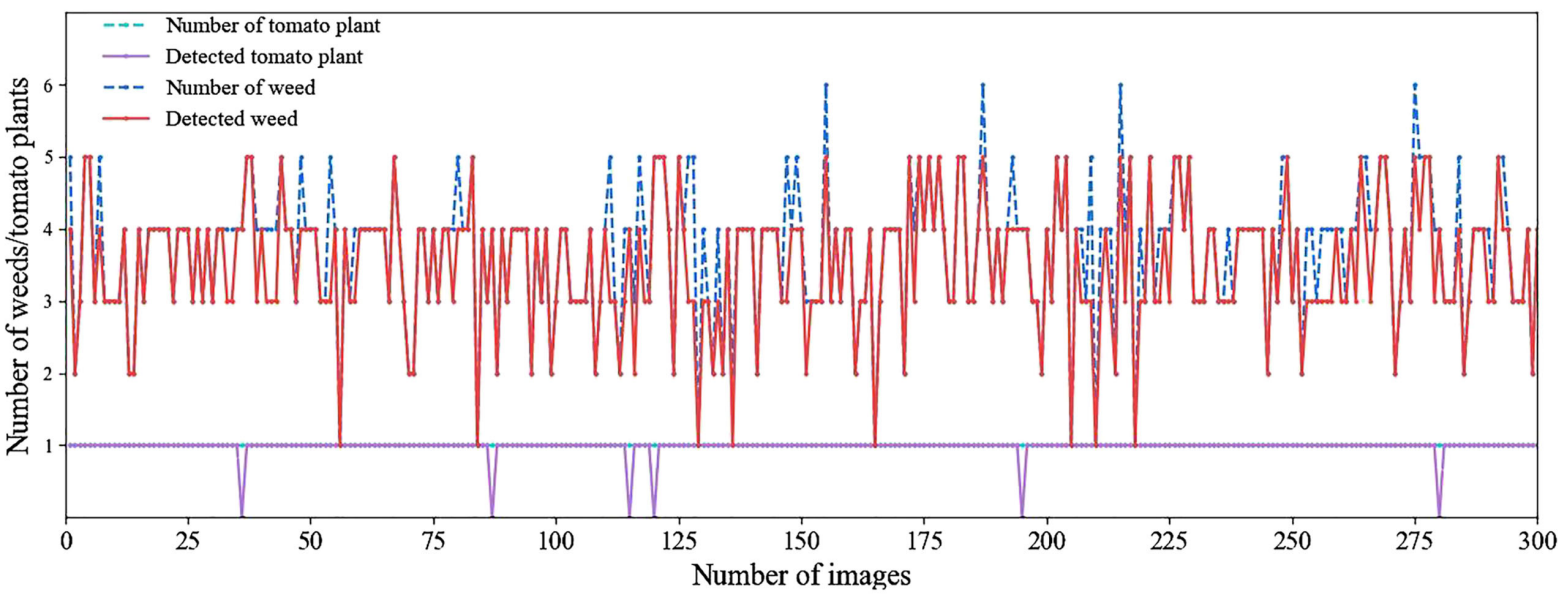
The weed and crop analysis per image: the number of weed/crop present in each image and the weed/crop in each image actually detected by the algorithm.

With the increase of the tolerance zone radius, the identification accuracy of all plants first increased then decreased. When the radius was 7.5 mm, the accuracy reached the maximum value of 95.19%. **Figure 8** shows data on the number of weeds and tomato plants present and detected in 300 images collected based on a circular tolerance zone with a radius of 7.5 mm. Among the 300 tomato plants and 1114 weed plants used in this experiment, a total of 294 tomato plants and 1052 weed plants were successfully located, which demonstrated that the system proposed in this study can effectively distinguish weeds from tomato plants.

## 4 Discussions

In this study, color mark sensors and cameras were combined in a pioneering way for plant detection. The development of an integrated sensing system that detects plant labels and calculates their location is the main innovation of this study. In order for the developed system to reliably classify different plants, the parameters of the color marker sensors and plant tags need to be determined, which is very important and necessary. The tomato plants were labeled using plant labels to give them a machine-readable signal. Thus, the algorithm to distinguish tomato plants from another unlabeled plant was simplified to improve the efficiency of computer execution. The system successfully located tomato and packchoi plants in real-time by using integrated sensing technology.

**Figure 9:**
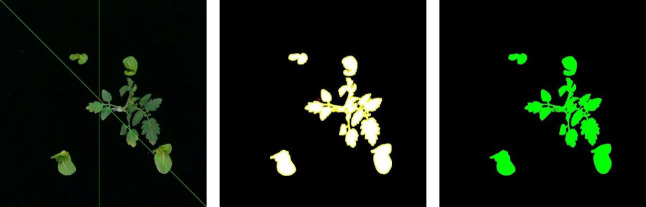
Misclassification due to low camera frame rate.

**Figure 10:**
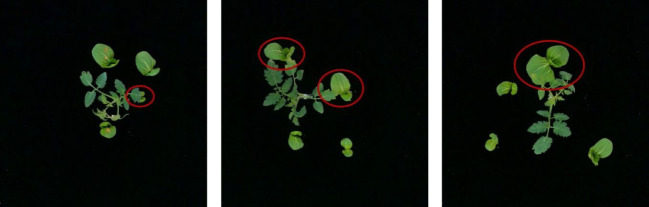
Weeds too close to tomato plants (red circles).

**Figure 11:**
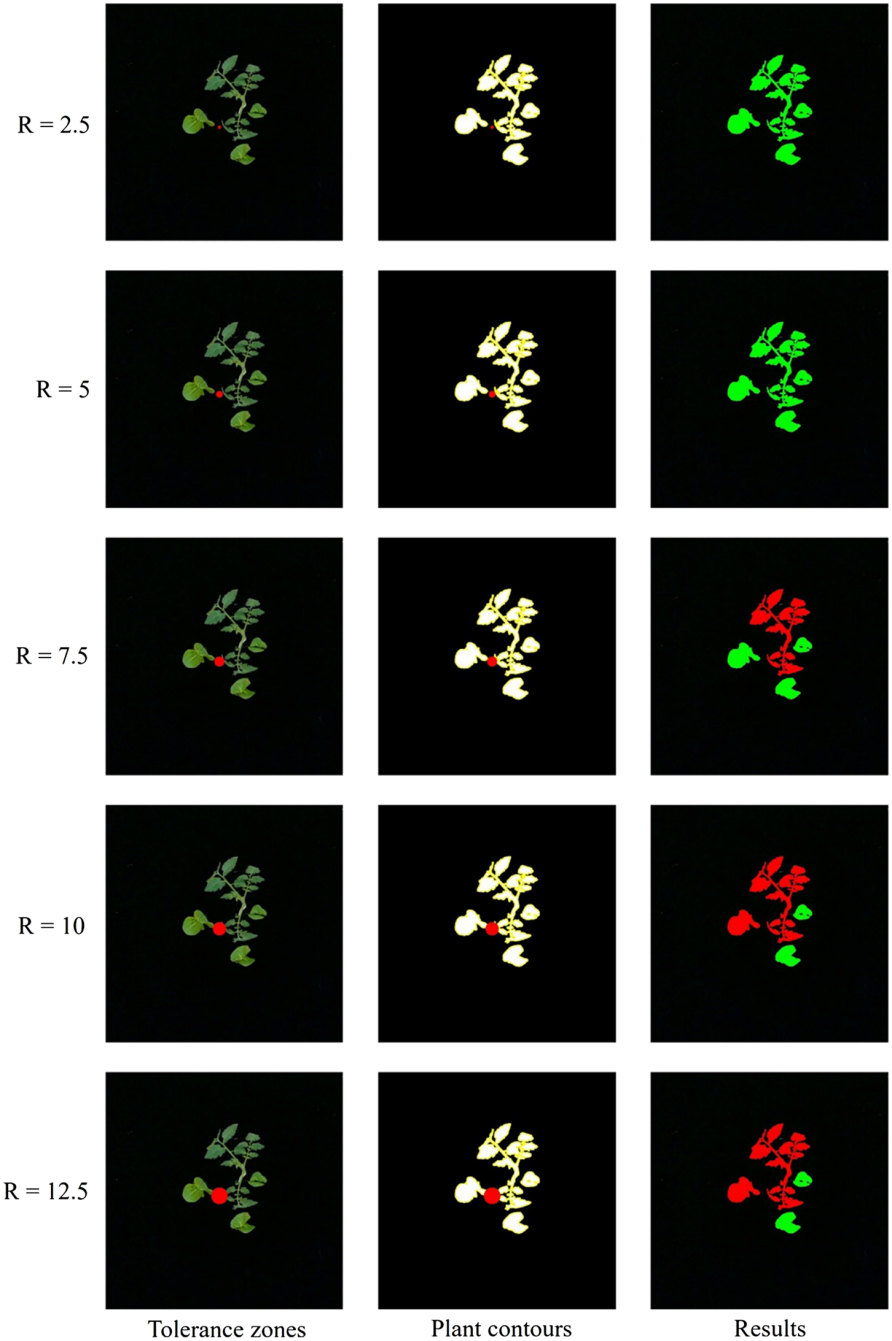
Five sizes of circular tolerance zones (red dots).

However, there are also a few plants that are not correctly localized by this system. The tomato plants were classified as weeds probably due to the low frame rate of the camera used in this system. After detecting a plant label, the next frame in the video stream was acquired by the computer as the image for calculating the plant location. Thus, the camera shutter did not open at the same moment as the sensor detected the plant labels in this image. The desynchronization between the camera shutter and the sensor caused incorrect predicted position of the plant label, as shown in **Figure 9**. In the future, high-speed cameras should be considered to improve the accuracy of plant classification. The errors introduced by the low frame rate of the camera and the delay of the command transmission were compensated by tolerance zones. The suitable size of tolerance zone facilitates the system to classify weeds and tomato plants precisely, as shown in **Figure 10**. In addition, weeds incorrectly classified as tomato plants possibly due to the occlusion caused by the tomato leaves and stem or weeds located too close to the tomato plants, as shown in **Figure 11**. In the future, an algorithm for segmenting overlapping weeds and tomato plants in the image should be developed to improve the accuracy of weed detection.

The color mark sensor selected in this study distinguished the target from other objects by using the difference in their reflection of red light. Based on this photometric principle, the color mark sensor showed different detection capabilities for objects of different colors or materials (Yang et al., 2008). Due to significant optical differences, the red light emitted by the color mark sensor can hardly be reflected by the stems of tomatoes and weeds, while it can be reflected by special plant labels. Thus, the plant labels were more easily detected by color mark sensors. Further, the weed stem is slightly lighter in color than the tomato stem, so it can reflect more red light. This might be the reason for the lower recognition accuracy of red and white plant labels when weed stem was used as a reference. When the tomato stem was used as the reference, the white plant labels were more readily detected than red and green plant labels. The reason for this may be that white labels reflect any wavelength of red light, while tomato stems reflect little red light. Although red labels can reflect red light, the wavelength range of the red light reflected did not exactly match the wavelength range of filter in the sensor, so only a portion of the red light reflected by the red labels received by the photosensitive element of the color mark sensor (Bayer-Krucsay, 1937). There was no significant difference in the light reflected by the green label and the green tomato stem, thus the color mark sensor could not distinguish between them.

A comparative analysis of the various weed and crop classification systems that have been developed are presented in **Table 3**. Comparison of crops, sensors, methods, detection speed and accuracy was performed to evaluate the performance of this system. Although only tomato plants were studied in this paper, the methods proposed in the study are applicable to most of the crops that need to be transplanted. Furthermore, although the studies presented in **Table 3** targeted different crops, they all used computer vision to identify tomato and packchoi plants. Thus, it is of great interest to compare the results of the present study with them.

**Table 3:** Comparison of various weed and crop classification method.

Study	Sensors	Crop	Method	Speed (ms f^-1^)	Accuracy (%)	
					Weed	Crop
Lee et al. (1999)	Color camera	Tomato	Machine vision	344	47.6	75.8
Lamm et al. (2002)	RGB, 3-CCD scan camera	Cotton	Machine vision	160	88.8	78.7
Ma et al. (2019)	Canon IXUS 1000 HS camera	Rice	SegNet	604	93.9	93.6
Garcia-Santillan and Pajares (2018)	Panasonic DMC-SZ8 color camera	Maize	On-line discrimination by Mahalanobis distance	280	91.8	n/a
Raja et al. (2020b)	RGB Basler 5 MP camera	Lettuce	Machine vision	160	83.74	99.75
Proposed method	Color mark sensor & camera	Tomato	Machine vision	30	98.00	94.44

The performance of the system is mainly reflected in speed and detection accuracy. Lee et al. (1999) earlier proposed a Bayesian-based classifier to classify tomato plants and weeds, which achieved a speed of 344 *ms f* ^-1^. However, its accuracy was low, with only 47.6% of the weeds and 75.8% of the crops being successfully classified. Lamm et al. (2002) developed a machine vision system to classify crops and weeds in commercial cotton fields based on their sizes, which improved the weed identification accuracy. Bakhshipour and Jafari (2018); Ma et al. (2019) proposed a weed detection method based on the SegNet semantic segmentation method and achieved a high accuracy rate (93.9% for weeds and 93.6% for crops), but the speed of their method was slow (604 *ms f* ^-1^). A method for distinguishing weeds in maize fields based on Mahalanobis distance was proposed by Garcia-Santillan and Pajares (2018). The method achieved an accuracy of 91.8% and a processing speed of 280 *ms f* ^-1^, but this method must obtain crop row information in advance. Then, Raja et al. (2020b) developed a machine vision system based on crop signaling technology to detect weeds in lettuce rows. The system achieved 83.74% weed detection accuracy and 99.75% crop detection accuracy at a processing speed of 160 *ms f* ^-1^. The crop classification accuracy and speed of this system met the requirements of real-time weeding, but the weed classification accuracy of this system was low. The method proposed in this study achieved the highest weed detection accuracy (98.00%) and the second highest crop detection accuracy (94.44%), which proves the effectiveness of the method. In addition, the system established in this study takes only 30 ms to process an image, which is much faster compared to the existing systems that take at least 160 ms to process an image.

## 5 Conclusions

An automatic real-time localization system was developed, which successfully located tomato and packchoi plants using integrated sensing techniques based on the crop signaling. With the plant labels applied to tomato plants, the information acquired by a color camera and six color marker sensors integrated by the developed image recognition algorithm. The results demonstrated that tomato stem was a reliable reference for higher accuracy. The detection accuracy of the white physical plant label was significantly higher than that of the red label and the green label. The lower part of the label was identified with the highest accuracy compared to the upper and middle part of the white physical plant label. The topical marker directly applied to the lower part of the plant stem can be more readily detected than the physical plant label. The combination of six color mark sensors is the best solution for detecting plant labels. A tolerance zone with a radius of 7.5 mm maximizes the accuracy of plant classification. Based on the established systematic method, the identification accuracy of tomato plants was 98.00%, and the accuracy of weeds was 94.44%. In addition, the system took only 30ms to process an image, which was faster than existing detection systems.Therefore, the system developed in this study had strong performance for weed and tomato identifications, which could provide prescription maps for weed control in transplanted vegetables at a faster rate.

## Data availability statement

The raw data supporting the conclusions of this article will be made available by the authors, without undue reservation.

## Author contributions

W-HS supervised this study, conceived the general idea, provided directions for the system development and revised the manuscript. J-LL developed the system, conducted experiments and analysed the data. W-HS, H-YZ and J-LL drafted the manuscript. W-HS and YP revised the manuscript and provided suggestions for the system development. All authors contributed to the article and approved the submitted version.
